# White Globules in Basal Cell Carcinoma: A Dermoscopic Sign With Preoperative Implications

**DOI:** 10.5826/dpc.1101a103

**Published:** 2020-01-29

**Authors:** Alessandra Pagnoni, Sabine Giroud, Angeliki Koulouri, Daniel Hohl, Olivier Gaide

**Affiliations:** 1 Department of Dermatology and Venereology, Lausanne University Hospital, CHUV, Lausanne, Switzerland

**Keywords:** basal cell carcinoma, MAY globules, optical coherence tomography, dermoscopy

## Introduction

Basal cell carcinoma (BCC) is the most frequent malignant tumor. Although usually indolent, specific subtypes (morpheaform, infiltrative, micronodular, and basosquamous) are associated with higher morbidity and recurrence rates [1]. Dystrophic calcifications (present in 10%–20% of BCCs) are mostly found in aggressive variants. Recognizing the dermoscopic signs of more invasive BCCs has important therapeutic implications.

Recently, a new dermoscopic criterion of higher-grade BCCs has been suggested: “multiple aggregated yellow-white (MAY) globules,” visible with both polarized and nonpolarized light [2]. Our case report emphasizes the importance of identifying these yellow-white globules for margin delineation before excision.

## Case Presentation

**Figure 1:**
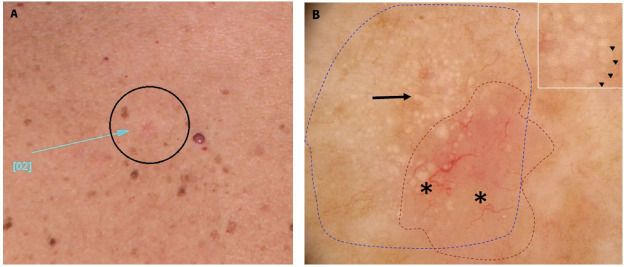
Multiple aggregated yellow-white globules (MAY globules) in calcifying micronodular basal cell carcinoma (BCC), preoperative: (A) Clinical image shows a faint pink macule (black circle). (B) Dermoscopy (×20, Medicam 800HD, FotoFinder) shows a pinkish area with arborizing telangiectasia (asterisks) suggestive of BCC (red dotted line corresponds to the site of initial excision). MAY globules (arrow, magnified in inset, arrowheads) are seen extending onto the upper and left side of the image (blue dotted line) that was included in the re-excision of the residual BCC).

**Figure 2:**
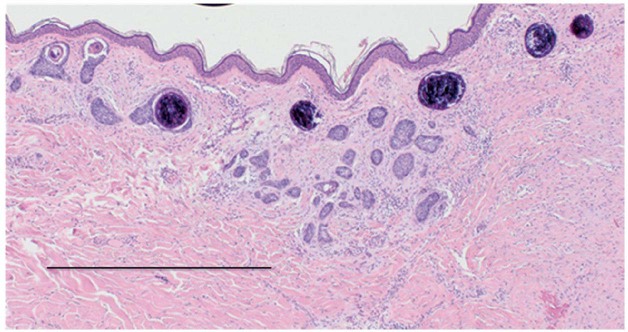
Histopathology of calcifying micronodular basal cell carcinoma (BCC; bar = 1 mm): Basaloid tumoral cells organized in small lobules in the superficial and mid-dermis; some of the lobules in the superficial dermis contain intratumoral calcifications (H&E, ×4).

A 65-year-old woman presented with an inconspicuous flat lesion on her back (Figure 1A). Dermoscopy revealed a few arborizing vessels and a pinkish area, suggestive of nodular BCC. More striking, however, was the presence of numerous well-defined yellow-white globules of unknown nature, starting within the telangiectatic area but spreading well beyond the known BCC criteria (Figure 1B). These structures were equally visible with polarized and nonpolarized dermoscopy. The initial excision, limited to the site with atypical vessels and pinkish area (red line in Figure 1B), revealed a calcifying micronodular BCC (Figure 2). The excision, however, was incomplete along the side of the yellow-white globules.

**Figure 3:**
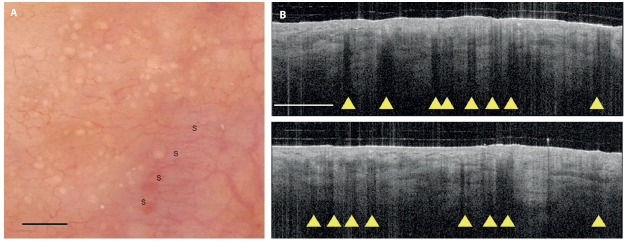
Correlation of optical coherence tomography (OCT) with dermoscopy (bar = 1 mm): (A) Dermoscopy (×20, Medicam 800HD, FotoFinder) after the first excision. MAY globules are still present beyond the scar (S). (B) OCT after the first excision. Vertical sections with hyporeflective cones correspond to MAY globules (yellow triangles).

Before reexcision, we complemented the dermoscopic examination (Figure 3A) with optical coherence tomography (OCT) imaging. OCT showed weakly refractile ovoid aggregates in the dermis, consistent with micronodular BCC (Figure 3B). It also showed peculiar dermal hyporeflective vertical columns that were probable shadows of yellow-white globules. In fact, the number and size of these structures matched that of yellow-white globules in dermoscopy, and of calcified nests and keratin cysts in histopathology.

The area with residual yellow-white globules was re-excised, and histopathology confirmed both the diagnosis of calcifying micronodular BCC and total excision.

## Conclusions

The structures identified in this report as yellow-white globules were very recently recognized as a new dermoscopic criterion, defined as “multiple aggregated yellow-white (MAY) globules” [2]. These are different from milia-like cysts and from shiny white strands and blotches since they are observed with both nonpolarized and polarized light. Navarrete-Dechent et al observed this feature in 21% of nonpigmented BCCs and in only 0.8% of other nonpigmented lesions, with a specificity of 99% for BCC [2]. Additionally, MAY globules were often associated with high-grade BCC subtypes, while it was never observed in superficial BCC. Our OCT and histopathologic findings were also consistent with their observations, namely, vertical optical shadows in OCT and dystrophic calcifications in histopathology.

Calcifications as well as micronodular histopathologic changes are associated with aggressive subtypes of BCC. Although we are reporting only on 1 patient, we believe that the identification of yellow-white/MAY globules has important management implications: first, it may alert to a more aggressive type of cancer, leading to different therapeutic strategies, and second, it may help in the preoperative delineation of tumor margins, reducing the frequency of re-excisions.
